# Giant photothermoelectric effect in silicon nanoribbon photodetectors

**DOI:** 10.1038/s41377-020-00364-x

**Published:** 2020-07-14

**Authors:** Wei Dai, Weikang Liu, Jian Yang, Chao Xu, Alessandro Alabastri, Chang Liu, Peter Nordlander, Zhiqiang Guan, Hongxing Xu

**Affiliations:** 1 School of Physics and Technology, Center for Nanoscience and Nanotechnology, and Key Laboratory of Artificial Micro- and Nano-structures of Ministry of Education, Wuhan University, Wuhan, 430072 China; 2 Department of Physics and Astronomy, Department of Electrical and Computer Engineering and Laboratory for Nanophotonics, Rice University, Houston, TX 77005 USA; 3 The Institute for Advanced Studies, Wuhan University, Wuhan, 430072 China

**Keywords:** Optics and photonics, Electronics, photonics and device physics

## Abstract

The photothermoelectric (PTE) effect enables efficient harvesting of the energy of photogenerated hot carriers and is a promising choice for high-efficiency photoelectric energy conversion and photodetection. Recently, the PTE effect was reported in low-dimensional nanomaterials, suggesting the possibility of optimizing their energy conversion efficiency. Unfortunately, the PTE effect becomes extremely inefficient in low-dimensional nanomaterials, owing to intrinsic disadvantages, such as low optical absorption and immature fabrication methods. In this study, a giant PTE effect was observed in lightly doped p-type silicon nanoribbons caused by photogenerated hot carriers. The open-circuit photovoltage responsivity of the device was 3-4 orders of magnitude higher than those of previously reported PTE devices. The measured photovoltage responses fit very well with the proposed photothermoelectric multiphysics models. This research proposes an application of the PTE effect and a possible method for utilizing hot carriers in semiconductors to significantly improve their photoelectric conversion efficiency.

## Photothermoelectric effect in silicon: a giant leap from light to electricity

Silicon nanoribbons can achieve a three to four orders of magnitude increased photovoltage response by photothermoelectric effect, relative to previously developed low-dimensional materials, offering greatly improved photoresponse, and practical application for on-chip photodetectors or power supplies. In the photothermoelectric effect, photoelectric conversion is achieved by the optical-induced temperature difference in the carrier system. The photothermoelectric effect is expected to utilize the hot carrier energy which is wasted in the traditional photoelectric conversion. The past reported photothermoelectric effect was in low-dimensional materials, such as carbon nanotubes, III–V semiconductor nanowires, dichalcogenide materials, and graphene. Researchers in China and the USA, led by Zhiqiang Guan and Hongxing Xu at Wuhan University, China, extended this effect to silicon nanomaterials. The successful observation of photothermoelectric effect in silicon relies on the low doping concentrations and nanometer size of the silicon nanoribbon. Ohmic electrode contact is also important to reveal the photothermoelectric effect instead of photovoltaic effect. This innovation greatly expands the practical application by the photothermoelectric effect and utilizes the hot carrier energy thus improve the photoelectric conversion efficiency. It could lead to more efficient light-powered systems, ranging from photo-detecting devices to solar cells.

## Introduction

Photoelectric energy conversion is a green energy conversion method applicable to energy and information devices. It has been reported that, in practice, up to 40% of the thermodynamic loss that occurs in photoelectric energy-conversion devices is a result of carrier thermalization loss and poor light absorption^1^. A key aspect of light–electricity conversion is the utilization of the thermal energy released during the relaxation of photogenerated hot carriers. An approach using the photothermoelectric (PTE) effect, in which an electrical signal is generated in response to the material thermoelectric effect and the temperature difference in the carrier system caused by incident light irradiation, has recently emerged. The PTE effect can utilize the energy of warm carriers and is thus expected to improve the photon responsivity of photodetectors and the energy conversion efficiency of solar cells^2^.

Work on the PTE effect, which originates from the difference in temperature between the decoupled carriers and the lattice, has significantly progressed in recent years. This effect usually occurs in nanomaterials because of the inefficient interaction of phonons with the carriers, especially in many low-dimensional nanomaterials. In a study of the PTE effect in graphene, photogenerated hot electrons played an important role in dual-gated graphene p-n junction devices^3^, which caused the photoresponse to exceed that of the photovoltaic (PV) effect in a graphene p-n junction. The cooling time of hot electrons was estimated to be 100 ps from the photocurrent profile, keeping the carrier temperature much higher than the lattice temperature. Moreover, the PTE effect has been reported in a wide range of materials, including carbon nanotubes^4–9^, III–V semiconductor nanowires^10^, and two-dimensional materials (e.g., graphene^3,11–13^, dichalcogenide materials^14,15^, and black phosphorus^16^). However, the photoresponse in low-dimensional materials caused by the PTE effect is low because of poor optical absorption. Practical PTE photodetectors are also difficult to use because of the immature fabrication methods used to produce low-dimensional materials.

In this study, the PTE effect was observed in lightly doped p-type silicon (Si) nanoribbons using scanning photocurrent microscopy (SPCM), and the effect was simulated by photothermoelectric multiphysics models. Successful observation of the PTE effect relied on suitable doping, the nanometer size of the Si nanomaterial, and the ohmic electrode contact. The open-circuit photovoltage responsivity reached 10^5^ V W^−1^ under weak irradiation with a 633-nm laser and was 3-4 orders of magnitude higher than that of previously reported devices using the PTE effect.

## Results

**Fig. 1:**
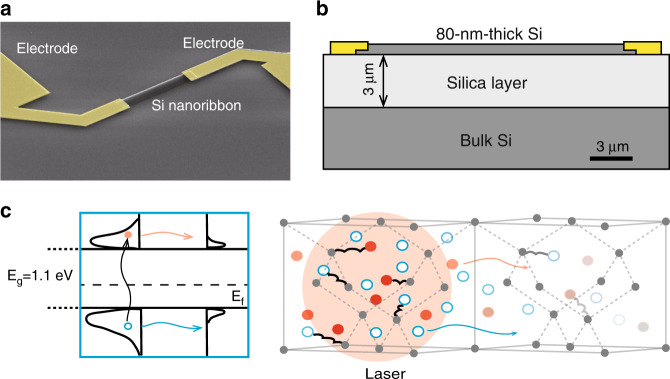
False-color scanning electron microscopy image, structural diagram of the device, and schematic of micromechanisms of the PTE effect. **a** False-color scanning electron microscopy image of the device. **b** Structural diagram of the device cross section. The length and width of the Si nanoribbon were 18 and 2.75 μm, respectively. The electrode was Au. **c** Schematic of the hot carrier generation in the Si band diagram (left) and their dynamics in a Si nanoribbon (right). Electron–hole pairs were generated using laser irradiation on the left part and diffused from the hot region to the far end. The hollow blue circles and solid red dots represent holes and electrons, respectively. The red and blue lines represent diffusion of the electrons and holes, respectively. The color intensity of the electrons represents kinetic energy. The large gray solid dots are the projected Si atoms in the diamond crystal structure.

Si nanoribbon photodetectors were fabricated on Si-on-insulator wafers (3-μm-thick SiO_2_ layer) with a p-type top Si layer (the doping concentration, 1.72 × 10^18^ m^−3^, was estimated from the measured electrical sheet conductance). A pseudocolor scanning electron microscopy image and a cross section diagram of the device are shown in Fig. 1a, b, respectively. The fabrication process is described in the “Materials and methods” section. The Si nanoribbon was 18 μm long, 2.75 μm wide, and 80 nm thick. The length and width of the nanoribbons were chosen based on the scanning range, step size, and laser spot size in SPCM. Gold (Au) and lightly doped p-type Si formed an ohmic contact, as the work function of Au (−5.1 eV) is lower than the Fermi level of lightly doped p-type Si (−4.74 eV). Lightly doped p-type Si was used because its Seebeck coefficient showed anomalous behavior with increasing temperature^17^. The thickness of the Si (80 nm) was less than the phonon mean free path, estimated to be 300 nm at 300 K^18^.

A one-dimensional transport model of the PTE effect is shown in Fig. 1c. Because the thickness of the Si was much smaller than the optical absorption depth (3.45 μm at a wavelength of 633 nm), the change in the carrier concentration in this direction was negligible. When the 633-nm focused laser irradiated the left end of the Si nanoribbon, many electron–hole pairs were generated at the irradiated position. The hot carriers were generated within hundreds of femtoseconds of carrier–carrier scattering^19^. The hot carriers then scattered with the optical phonons and approached the quasiequilibrium state within hundreds of picoseconds, and the carrier temperature was higher than that of the lattice. For Au/Si ohmic contact PTE effect devices, the main contribution to the photocurrent is the photogenerated hot carriers diffusing from the excitation (hot) region to the far end, driven by the carrier temperature and concentration gradient. Because p-type Si was used, the holes contributed more to the thermoelectric process, forming a net current flowing from the excitation region to the far end (*j* = −*σS*∇*T*, where *σ* is conductivity, and *S* is the Seebeck coefficient; *S* > 0 for p-type Si, because hole diffusion is dominant). The measurable photocurrent/photovoltage of the PTE effect also benefitted from the high Seebeck coefficient of the lightly doped p-type Si (measured value 9.9 mV K^−1^). For comparison, a PV effect device was also studied in this work. The open-circuit voltage depends upon the built-in voltage and how far the Fermi levels must change for the internal diffusion current to exactly balance the photocurrent to give an external zero current. The Fermi energy difference resulted in an induced electric field that separated the photogenerated electron–hole pairs^20^. The PV effect is commonly observed in p–n junctions or metal/semiconductor Schottky junctions. PTE and PV effects could be distinguished by the sign of the open-circuit voltage/short-circuit current from the scanning photovoltage/photocurrent microscopy^21^. Differentiating the PTE effect from the PV effect has been reported in black phosphorus^16^ and MoS_2_^22^.

**Fig. 2:**
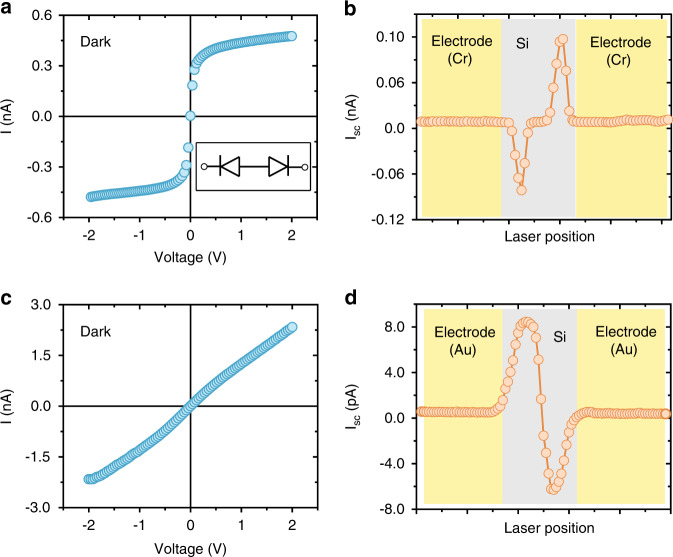
Measured *I–V* curves and scanning photocurrent of the ohmic/Schottky electrode contact device. The measured *I–V* curves of **a** the Schottky electrode contact device and **c** the ohmic electrode contact device without light irradiation. **b**, **d** The corresponding short-circuit photocurrent, *I*_sc_, scanned in space. The laser wavelength was 633 nm, and the power was 0.18 μW (3.67 W cm^−2^). The yellow and gray regions correspond to the electrode and Si nanoribbon, respectively.

The presence of the PTE effect in Si nanoribbon photodetectors was confirmed by the experimental results as follows. First, it was confirmed by SPCM of different electrode contact devices^10,21,23^. The key factor affecting the PV and PTE effects was the contact properties of the metal and Si. Possibly owing to the lack of annealing electrodes in the experiment, while most devices showed ohmic contacts because of the Au/Si contact, some devices showed Schottky contact behavior because of the chromium (Cr)/Si contact. Schottky contact is formed because the work function of Cr (−4.5 eV) is higher than the Fermi level of lightly doped p-type Si. Thus, it forms a barrier to the holes. The metal–semiconductor contact types for different devices were confirmed by their measured *I–V* characteristics. Figure 2a, c shows the *I–V* curves for two devices having different contacts. The *I–V* curves were measured under dark conditions over a voltage range from −2 to 2 V, which was sufficiently broad to verify the electrode contact type. For the sample having Schottky contacts, the *I–V* curve represented the response of two back-to-back series diodes, in which the current showed a symmetric reverse-bias-dominated response with external voltage. When the applied voltage was greater than 0.2 V, the gradually increasing leakage current could have been due to either the additional barrier height reduction upon applied voltage, such as the Schottky effect, or the increased number of thermally generated minority carriers in the larger depletion region at higher applied voltages^24^. For the device having ohmic contacts, a linear *I–V* curve was observed, as shown in Fig. 2c, indicating that there was no significant band bending effect.

Figure 2b, d shows the corresponding SPCM results for Schottky and ohmic devices. The short-circuit current, *I*_sc_, was measured under ambient conditions without external bias. The laser wavelength was 633 nm, and the spot diameter was 2.5 μm. For the Cr/Si Schottky contact devices, the maximum value of the photocurrent occurred near the electrode and at the edge of the Si nanoribbon with a negative photocurrent on the left side of the device and a positive photocurrent on the right side of the device, as shown in Fig. 2b. The direction of the photocurrent indicated a downward energy band bending in the device. These observations indicate electron–hole separation in the Schottky junction, meaning that the photocurrent was caused by the PV effect. However, for the ohmic device, the photocurrent scanning result was the opposite of that for the device with the Schottky junction. The reversed sign of the photocurrent can be attributed to the PTE effect, in which the current direction was determined by the carrier temperature gradient and the majority carrier diffusion. Thus, these results successfully differentiated the PV effect from the PTE effect for different Schottky/ohmic contacts.

**Fig. 3:**
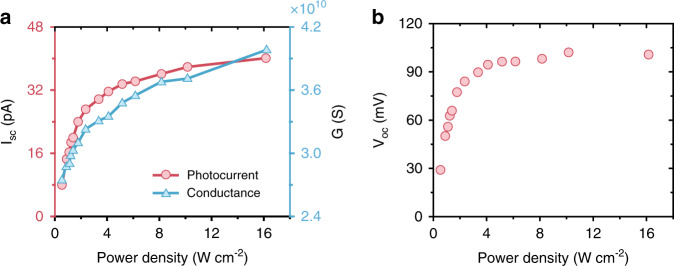
Measured dependence of the photoresponse and conductance of the ohmic device on the laser power density. **a** The measured dependence of the short-circuit current, *I*_sc_, and conductance, *G*, on the power density. The conductance was calculated using the slope of the *I–V* curve. **b** The measured dependence of the open-circuit voltage, *V*_oc_, on the laser power density. The 633-nm laser irradiated the left end of the Si nanoribbon.

Second, the PTE effect in Si nanoribbon photodetectors was evidenced by the large photovoltage, which would have been impossible from the thermoelectric effect caused by the small increase in lattice temperature. The increase in lattice temperature in the illuminated Si nanoribbon was estimated by both a COMSOL simulation and the reported photoinduced lattice temperature increase coefficient. The increase in lattice temperature in the Si nanoribbon in the irradiated region estimated by COMSOL was <0.01 K under an experimental illumination power density of 2 W cm^−2^. Figure S2 shows the temperature distribution simulated by the COMSOL heat module. A photoinduced lattice temperature increase coefficient in Si of 0.88 K at 1 W cm^−2^ was reported previously^25^. Considering that the optical absorption efficiency of the present Si nanoribbon device was only 1%, the photoinduced increase in lattice temperature was <0.02 K at 2 W cm^−2^. From the two estimations, it was clear that the increase in lattice temperature in the present Si nanoribbon was <0.02 K. Thus, the thermoelectric voltage driven by the lattice temperature difference was <0.2 mV, as evaluated with a Si Seebeck coefficient of 9.9 mV K^−1^. This is much smaller than the experimental value of the photovoltage response (~80 mV at 2 W cm^−2^, as shown in Fig. 3b). Therefore, the experimental photovoltage response cannot be attributed to the thermoelectric voltage driven by the lattice temperature difference. Instead, it originated from the PTE effect driven by the carrier temperature difference.

To further study the photoresponse caused by the PTE effect, the dependence of the photoresponse (i.e., open-circuit voltage *V*_oc_ and short-circuit current *I*_sc_) on the laser power density was measured and is shown in Fig. 3. The laser spot was at one end of the ohmic device. The dependence of the conductance on the laser power density was also measured from the slope of the *I–V* curve and is plotted in Fig. 3a. *V*_oc_ showed a nonlinear increase and saturation at large laser power density values. For laser power densities > 4 W cm^−2^, *V*_oc_ gradually approached a saturation value of ~100 mV. When *V*_oc_ approached the saturation value, the conductance and *I*_sc_ of the device still increased with the laser power density but tended to saturate. Considering that the change in the carrier mobility with carrier concentration is <1% when the carrier concentration does not exceed 10^22^ m^−3^, according to the mobility formula in COMSOL, the increase in the conductivity, *σ*_*e*_ = *qμ*_*e*_*n*, where *q* is the charge, *μ*_*e*_ is electron mobility, and *n* is the electron concentration, indicates an increase in carrier concentration. Hence, the saturation behavior of *V*_oc_ is likely to be explained by the saturation of the carrier temperature with increasing laser power density, as corroborated by the models described below. The photovoltage responsivity in the Si nanoribbon ohmic device using the PTE effect was observed to be as high as 10^5^ V W^−1^ for weak illumination at 633 nm. The photovoltage responsivity of this device was 3-4 orders of magnitude higher than the reported photovoltage responsivity in the visible band caused by the PTE effect in previous devices^4,6,14,26^.

To understand the PTE effect in Si nanoribbons and the saturation behavior of *V*_oc_ with increasing laser power density, a photothermoelectric multiphysics model was established using the finite element software COMSOL, including light absorption, photogeneration and recombination of carriers, charge transport, and a two-temperature model of the carrier and lattice system. The model of the steady-state distribution includes three equations: the dynamic balance of the carrier number density, the heat–energy balance, and the electric potential balance (see Supporting Information). In the model, the lattice temperature was set to room temperature, and the model only calculated the heat–energy balance equation for the carrier system.

**Fig. 4:**
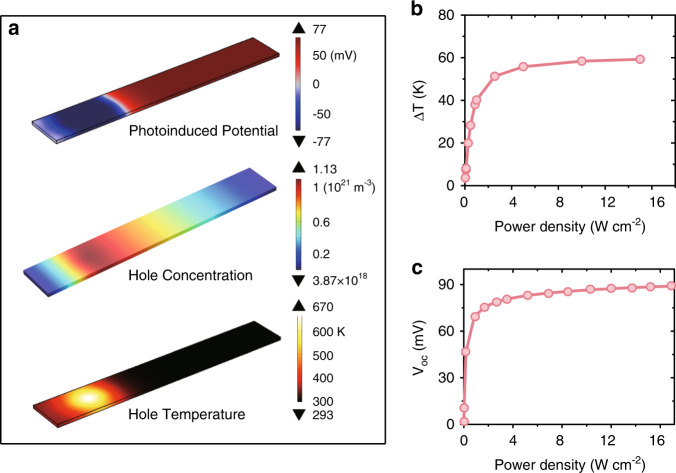
Simulation results of the PTE effect in Si nanoribbons. **a** Simulated spatial distribution of voltage, hole concentration, and hole temperature in the Si nanoribbon at 16.2 W cm^−2^ laser power density. **b** Simulated dependence of the carrier temperature difference (Δ*T*) between the two ends of the nanoribbon on laser power density. **c** Simulated dependence of the open-circuit voltage, *V*_oc_, on the laser power density.

The multiphysics model confirmed the carrier temperature gradient and the PTE effect in the Si nanoribbons with ohmic contact. The simulation results are shown in Fig. 4. The electron/hole temperature distribution was formed in the device when the laser illuminated the left side of the Si nanoribbon. The hole temperature distribution is shown in Fig. 4a. The hole temperature reached a maximum of 670 K at the center of the laser spot and maintained a temperature difference of 55 K between the two ends of the Si nanoribbon (Fig. 4b). The concentration distribution of holes had a maximum value of 10^21^ m^−3^ (see Fig. 4a, middle), and the voltage distribution in the open-circuit condition is shown at the top of Fig. 4a. The simulated spatial distributions of electron concentration and electron temperature in the Si nanoribbons are shown in Fig. S3. The simulated dependence of *V*_oc_ on laser power density is shown in Fig. 4c. There is good agreement with the experimental results. The saturation behavior of the *V*_oc_ curves was a result of saturation of the carrier temperature difference between the two ends of the Si nanoribbons as laser power density increased (Fig. 4b). A possible reason for this is that the heat capacity and thermal conductivity of the carriers increase rapidly with increasing carrier temperature, as shown in Fig. S4.

**Fig. 5:**
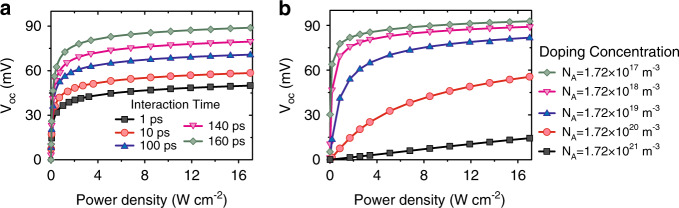
Simulated photoresponse of the optimized device. The simulated dependence of *V*_oc_ on the laser power density **a** for different carrier–lattice interaction times *τ*_*c*_ and **b** for different doping concentrations *N*_A_.

Steady-state decoupling between the carrier and lattice temperatures in the Si nanoribbon occurred because of suppressed acoustic phonon scattering and weakened coupling between the carrier and the lattice system, as shown in the Supporting Information. The carrier–lattice interaction time had a significant effect on the temperature difference between the two ends of the Si nanoribbons, and the saturation value of the open-circuit voltage was affected, as shown in Fig. 5a. The dependence of the experimental *V*_oc_ on the laser power density was confirmed by the multiphysics model by setting the carrier–lattice interaction time *τ*_*c*_ = 160 ps, as shown in Figs. 3b and 4c. The value of *τ*_*c*_ was consistent with that of Si nanowires^27,28^, showing that the nanoscale limited the acoustic phonon scattering and suppressed the carrier–phonon interaction. This mechanism is similar to the one that describes the steady-state carrier temperature difference of 190 K in III–V semiconductor nanowires^29^. Therefore, a useful development will be to optimize the nanostructure design to suppress phonon scattering and facilitate the PTE response.

An optimized doping concentration also contributes to the giant PTE photovoltage response, as in carbon nanotube p–n junctions^6^. A multiphysics model was used to calculate the dependence of *V*_oc_ on the laser power density for different doping concentrations in the Si nanoribbons, as shown in Fig. 5b. The optimized doping concentration was the same as that in the Si-on-insulator wafers used in the experiment. The doping concentration also affected the slope of the *V*_oc_ vs. laser power density curve. Thus, a smaller doping concentration would cause a higher photoresponsivity while reducing the linear photoresponse working window.

The measured dynamic response of *I*_sc_ in the Si nanoribbon is shown in Fig. S5. The switching time of *I*_sc_ between the laser on and off states was ~120 ms, similar to previously reported PTE effects^30,31^. This is much slower than the time scale of the carrier temperature changes (nanoseconds). The main reason for this is the slow diffusion transport of the holes in the lightly doped p-type Si. This could be solved by ballistic transport in sub-10-nm-thick Si films. Another reason for the slow response could be the effect of charge trap states. This could be solved by surface passivation or fast charging and discharging with a gate voltage^32^.

## Discussion

Si nanomaterials are very promising candidates for use in practical photodetection applications. They have excellent photoelectric and thermoelectric properties^33,34^ and can be integrated on a large scale using mature complementary metal–oxide–semiconductor fabrication techniques. The fabrication method of the proposed device is fully controllable and completely compatible with existing mature semiconductor technology. In addition, previous studies of hot carriers in Si primarily focused on the transient temperature increase of carriers generated by short pulse lasers^35–37^. Subsequent realization of steady-state decoupling of the carrier temperature from the lattice temperature is crucial for the practical application of Si photodetectors based upon the PTE effect. Furthermore, it is possible to cooperatively utilize the PTE and PV effects by matching the Seebeck coefficient polarity to the energy-band bending direction. Plasmon-enhanced optical absorption is another strategy to enhance the PTE effect, as demonstrated in a previous work^38^.

Based on the curve of the open-circuit voltage *V*_oc_ dependence of the laser power density (Fig. 5b), the linear working region of *V*_oc_ and the change in *V*_oc_ with incident light power density can be adjusted by changing the doping concentration. A linear photoresponse working region extending over a wider range of light power densities can be realized by higher doping concentrations. On the other hand, the PTE photoresponse with a low doping concentration is more sensitive to a weaker laser power density. Therefore, this class of PTE photodetectors can be used under either high or low incident light power density by choosing proper doping concentrations.

In conclusion, a giant PTE effect caused by the steady-state carrier temperature difference was observed in lightly doped p-type Si nanoribbons. The open-circuit photovoltage responsivity caused by the PTE effect was 10^5^ V W^−1^ in the linear response region upon 633-nm wavelength illumination without an external bias voltage. This is 3-4 orders of magnitude higher than the PTE effects reported previously. This high photoresponsivity was achieved by forming an ohmic electrode contact that excludes the PV effect, nanometer-sized Si nanoribbons that suppress carrier–phonon interaction, and an optimized doping concentration. A photothermoelectric multiphysics model based on the finite element method was presented to clarify the PTE effect in Si nanoribbons. The PTE device can be used as a photodetector under strong or weak light conditions, depending on the doping concentration of Si. By combining the advantages of mature fabrication and integration techniques, the giant PTE effect, and successful enhancement strategies, this research proposes an application of the PTE effect and utilization of hot carriers in semiconductors and the optimization of photoelectric conversion efficiency.

## Materials and methods

### Fabrication procedure

The sample employed in this study was fabricated on an 8-in. Si-on-insulator wafer (Shenyang Silicon Technology Co. Ltd, China) with a top layer of Si (p-type, crystal orientation (100), thickness 220 nm, and resistivity 0.085−0.115 Ωm), a 3-μm middle layer of SiO_2_, and a 750-μm Si substrate. After metal alignment marks were made on the wafer via photolithography and thermal evaporation, the Si-on-insulator wafer was cut into small pieces and thoroughly cleaned using the RCA cleaning process, named for the company that invented it: Radio Corporation of America^39^. Next, the thickness of the top Si layer was reduced to 80 nm via reactive ion etching (RIE-150A, Tyrone Electronics, China, SF6, 200-sccm flow rate, 200-W RF power, 190-V bias voltage, 4.6-Pa vacuum, for 20 s) and washed using the RCA cleaning process. The Si nanoribbons were produced by e-beam lithography (Raith Elline Plus, Germany, 20 kV, 450-nm polymethyl methacrylate (PMMA), 200 μC cm^−2^) and reactive ion etching, whose parameters were the same as for the Si thinning process.

After removing the PMMA by immersion in hot acetone for 15 min and performing the RCA cleaning process, the metal electrodes were produced via e-beam lithography using an alignment mask, thermal evaporation, and photolithographic lift-off. The evaporated metal was 5-nm Cr (0.2 Å s^−1^) and 150-nm Au (0.5 Å s^−1^). The microfabrication process is shown in Fig. S1.

### Measurements

The doping concentration of the top Si was calculated using the measured sheet electrical conductance. The measured Seebeck coefficient, refractive index, and experimental conditions for SPCM were reported in a previous work^38^. The SPCM measurements were performed under ambient conditions without external bias. The laser wavelength was 633 nm, and the spot diameter was 2.5 μm. The thermal conductivity of the top Si layer and the SiO_2_ layer was measured using time-domain thermoreflectance^40^.

## Supplementary information

Supplementary Information

## Data Availability

The data supporting the findings of this study are available from the corresponding authors upon request.
