# Supplementary material for: Giant photothermoelectric effect in silicon nanoribbon photodetectors
Source: Light Sci Appl. 2020 Jul 14;9:120. doi: 10.1038/s41377-020-00364-x (PMC7360756; doi:10.1038/s41377-020-00364-x)
Supplement: Supplementary file 1 — Supplementary Information [file 41377_2020_364_MOESM1_ESM.docx]

Supporting Information for

# Giant Photothermoelectric Effect in Silicon Nanoribbon Photodetectors

**Wei Dai^1,#^, Weikang Liu^1,#^, Jian Yang^3,#^, Chao Xu^1^, Alessandro Alabastri^3^, Chang Liu^1^, Peter Nordlander^3^, Zhiqiang Guan^*,*^, Hongxing Xu^1,2,*^**

^1^School of Physics and Technology, Center for Nanoscience and Nanotechnology, and Key Laboratory of Artificial Micro- and Nano-structures of Ministry of Education, Wuhan University, Wuhan 430072, China

^2^The Institute for Advanced Studies, Wuhan University, Wuhan 430072, China

^3^Department of Physics and Astronomy, Department of Electrical and Computer Engineering and Laboratory for Nanophotonics, Rice University, Houston, Texas 77005, United States

**# Equal contributions * Corresponding authors**

**Correspondence:**

Hongxing Xu: hxxu@whu.edu.cn

Zhiqiang Guan: [zhiqiang.guan@whu.edu.cn](mailto:zhiqiang.guan@whu.edu.cn)

## Sample fabrication process


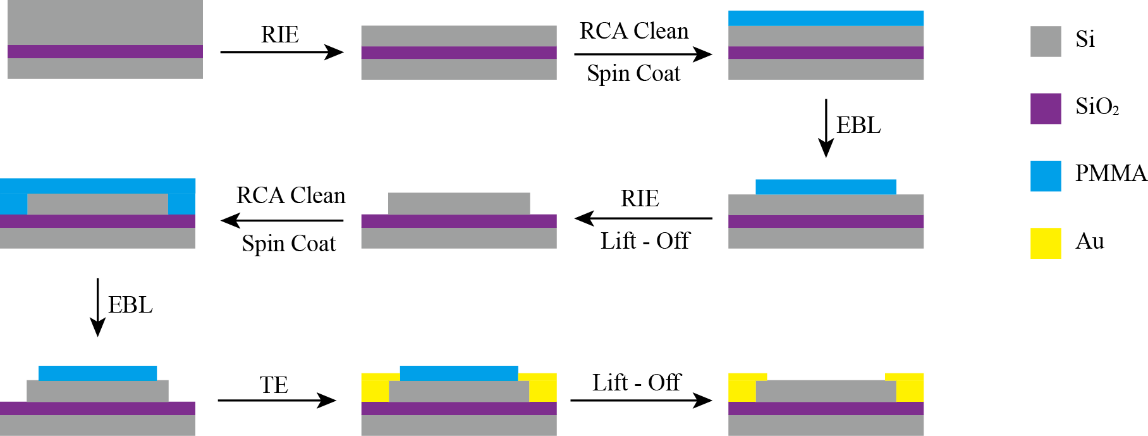


**Fig. S1.** The microfabrication process of the Si nanoribbon device. RIE: Reaction ion etching. EBL: Electron beam lithography. TE: Thermal evaporation.

## Simulation methods

The reflectance of the device was calculated by Fresnel’s law for the multilayer model. The refractive index of gold was from Babar and Weaver^1^. The refractive index of SiO_2_ was from Palik^2^. The refractive index of the p-type Si used the measured data from ellipsometry.

### Modeling of PTE Effect

The drift-diffusion model in the COMSOL Semiconductor Module was valid for the devices as the device length was more than several hundred nanometers. Approximations used in the model include: the energy band structure of Si was simplified as parabolic, the complexity of the energy band structure near the free energy surface and the crystal boundary were ignored, the doping effect in the Si from the metal contact was ignored, and the influence of trap states on the Si surface or caused by the SiO_2_ substrate was ignored.

### Calculation of the Open-circuit voltage $\mathbf{V}_{\mathbf{oc}}$

First, the carrier number balance equation in steady-state was established. The photogenerated electron–hole pairs and their recombination in Si nanoribbons under laser irradiation were considered. Laser radiation of wavelength $\text{λ}_{\text{0}}=\text{633 nm}$ has photon energy 1.96 eV, larger than the Si bandgap 1.12 eV. Therefore, single-photon absorption and free-carrier absorption^3^ were mainly considered. The modeling of the carriers included direct recombination, Auger recombination^3,4^, and impact ionization^3,5^. Two-photon absorption, photoelectron emission, and defect recombination in this model were ignored. The balanced equations of the electron and hole number in Steady State are:

$\frac{Q\left( \beta+\Theta n \right)}{hv}=\xi np+\gamma n^{3}-\delta n$ (S1)

$\frac{Q\left( \beta+\Theta p \right)}{hv}=\xi np+\gamma p^{3}-\delta p$ (S2)

Here the power density of optical absorption in the Si nanoribbons is:

$Q\left( r \right)=I_{0}\left( 1-R \right)\left[ 1-\exp\left( -\alpha z \right) \right]{\exp\left( -{r^{2}}/{r_{0}^{2}} \right)}/z$ (S3)

where $I_{0}$is the incident laser power density, *R* is calculated reflectance, $\alpha={4\pi Im\left( n \right)}/{\lambda_{0}}$ is the absorption coefficient, $n$is the refractive index of Si, $r_{0}=\text{1.25 μm}$ is the radius at the waist of the laser beam, the Si thickness $z=\text{80 nm}$, *n* and *p* are the electron and hole concentration respectively, $hv$ is photon energy, $\beta=\text{1.021×1}\text{0}^{\text{5}}\text{ }\text{m}^{\text{-1}}$ is the single-photon absorption rate, $\Theta\text{ = 5×1}\text{0}^{\text{-18}}(T_{c}/\text{300 K})\text{ c}\text{m}^{\text{2}}$ is the free carrier absorption rate^3^, $\xi$ is the direct recombination rate which needs to be calculated, $\gamma\text{ = 3.8×1}\text{0}^{\text{-}\text{43}}\text{ }\text{m}^{\text{6}}\text{ }\text{s}^{\text{-1}}$ is the Auger recombination rate, $\delta\text{ = 3.6×1}\text{0}^{\text{10}}\exp(\text{-}\text{1.5} E_{g}/k_{B}T_{c}) \text{s}^{\text{-1}}$ is the ionization rate, and $T_{c}$ is the carrier temperature. The values of the relevant parameters are given in the Table S1.

Second, the balance equation of the electric potential was established. For the $V_{\mathrm{oc}}$ calculation model, the equations are:

$\nabla\cdot\left( -\varepsilon_{r}\nabla V \right)=q\left( p-n+N_{D}-N_{A} \right)$ (S4)

$j_{e}=j_{h}=0$ (S5)

where $N_{A}=\text{1.72×1}\text{0}^{\text{18}}\text{ }\text{m}^{\text{-3}}$ is the p-type doping concentration, $n=N_{C}F_{\frac{1}{2}}\left( \eta_{e} \right)$, $p=N_{V}F_{\frac{1}{2}}\left( \eta_{h} \right)$, $N_{C}=\text{2}\left( \frac{m_{e}^{*}k_{B}T_{c}}{2\pi\hbar^{2}} \right)^{\frac{3}{2}}$, $N_{V}=\text{2}\left( \frac{m_{h}^{*}k_{B}T_{c}}{2\pi\hbar^{2}} \right)^{\frac{3}{2}}$. $\eta_{e}=\frac{E_{F}-E_{C}}{k_{B}T}$, $\eta_{h}=\frac{E_{V}-E_{F}}{k_{B}T}$, $E_{C}=-\left( V+\chi\right)$, $E_{V}=-\left( V+\chi+E_{g} \right)$, and $F_{j}$ is the order j of the Fermi-Dirac integral $F_{j}\left( \eta\right)=\frac{1}{\Gamma\left( j+1 \right)}\int_{0}^{\infty} \frac{\varepsilon^{j}}{1+\exp\left[ \varepsilon-\eta\right]}d\varepsilon$. As long as the carrier concentration is less than 10^23^ m^-3^, the bandgap narrowing effect can be neglected^6,7^. The current density is defined as:

$j_{e}=q\mu_{e}n\nabla V+\mu_{e}k_{B}T_{c}\frac{F_{\frac{1}{2}}\left( \eta_{e} \right)}{F_{-\frac{1}{2}}\left( \eta_{e} \right)}\nabla n-\mu_{e}nk_{B}\left[ \frac{3}{2}\frac{F_{\frac{1}{2}}\left( \eta_{e} \right)}{F_{-\frac{1}{2}}\left( \eta_{e} \right)}-\left( r+\frac{5}{2} \right)\frac{F_{r+\frac{3}{2}}\left( \eta_{e} \right)}{F_{r+\frac{1}{2}}\left( \eta_{e} \right)} \right]\nabla T_{c}$ (S6)

$j_{h}=q\mu_{h}p\nabla V-\mu_{h}k_{B}T_{c}\frac{F_{\frac{1}{2}}\left( \eta_{h} \right)}{F_{-\frac{1}{2}}\left( \eta_{h} \right)}\nabla p+\mu_{h}pk_{B}\left[ \frac{3}{2}\frac{F_{\frac{1}{2}}\left( \eta_{h} \right)}{F_{-\frac{1}{2}}\left( \eta_{h} \right)}-\left( r+\frac{5}{2} \right)\frac{F_{r+\frac{3}{2}}\left( \eta_{h} \right)}{F_{r+\frac{1}{2}}\left( \eta_{h} \right)} \right]\nabla T_{c}$ (S7)

which includes drift current and diffusion current driven by the concentration and the temperature gradient. $r=\text{1}/\text{2}$ corresponds to acoustic phonon scattering and was used in the model. $q$is the charge, $\mu_{e}$and $\mu_{h}$ are the electron and hole mobilities respectively, and $k_{B}$ is the Boltzmann constant. The influence of the lattice scattering and the carrier–carrier scattering on the carrier mobility was included. The subscripts *c*, *e*, *h*, and *l* denote carrier, electron, hole, and lattice, respectively.

Last, the heat–energy balance equation was established. The two-temperature model with carrier temperature and lattice temperature was used. The thermal radiation effect was ignored. Considering that the thickness of Si nanoribbons (80 nm) was much smaller than the attenuation depth of 633 nm wavelength laser radiation ($3.45 \mu m$), and the thermal conductivity of Si ($\text{131 }\text{W }\text{m}^{\text{-1}}\text{ }\text{K}^{\text{-1}}$) was much higher than that of silica ($1.38 \text{W }\text{m}^{\text{-1}}\text{ }\text{K}^{\text{-1}}$), the lattice temperature was assumed to have a uniform distribution in the z-direction. The model used the thermal conductivity of bulk Si as an upper limit and ignored anisotropy and the size effect of the thermal conductivity^8,9^. In this model, the lattice temperature was set to room temperature $T_{l}=\text{293.15 K}$ and the model only calculated the heat–energy balance equation of the carrier system^10^:

$\nabla\cdot\left( -\kappa_{c}\nabla T_{c} \right)=Q\left( \beta+\Theta n \right)-\gamma_{cl}\left( T_{c}-T_{l} \right)$ (S8)

The electron and hole temperatures were set as $T_{c}=T_{e}=T_{h}$. $\kappa_{c}=\frac{k_{B}^{2}T_{c}}{q}\left( \mu_{e}n+\mu_{h}p \right)$ is the effective thermal conductivity of carriers, $\gamma_{cl}=\frac{C_{c}}{\tau_{c}}$ is the coupling strength of the carrier-lattice system, where $C_{c}=\frac{3}{2}k_{B}\left( n+p \right)$ is the heat capacity of the carrier, and $\tau_{c}$ is the carrier-lattice interaction time.

The electrode contact was set as ohmic in the PTE effect model. The electrical boundary condition was that the current density at both ends is 0. The thermal boundary condition was that the net heat flux at the Si/SiO_2_ interface and the Si/electrode interface is 0 ($\nabla\cdot q=0$). The initial temperatures for the lattice and the carriers were 293.15 K. The device parameters were set according to the experimental values. The equations S1, S3, and S7 were solved iteratively until convergence to get the voltage V and carrier temperature $T_{c}$.

#### Calculation of the Short-circuit current $\mathbf{I}_{\mathbf{sc}}$

The balanced equations of the carrier number in the steady-state are:

$\nabla\cdot j_{e}+\frac{Q\left( \beta+\Theta_{e}n \right)}{hv}=\xi np+\gamma n^{3}-\delta n$ (S9)

$\nabla\cdot j_{h}+\frac{Q\left( \beta+\Theta_{h}p \right)}{hv}=\xi np+\gamma p^{3}-\delta p$ (S10)

The heat–energy balance equation is:

$\nabla\cdot\left( -\kappa_{c}\nabla T_{c}+ST_{c}j_{c} \right)=Q\left( \beta+\Theta n \right)-\gamma_{cl}\left( T_{c}-T_{l} \right)$ (S11)

*S* is the Seebeck coefficient:

$S_{e}=\frac{k_{B}}{q}\left[ \frac{3}{2}\frac{F_{\frac{1}{2}}\left( \eta_{e} \right)}{F_{-\frac{1}{2}}\left( \eta_{e} \right)}-\left( r+\frac{5}{2} \right)\frac{F_{r+\frac{3}{2}}\left( \eta_{e} \right)}{F_{r+\frac{1}{2}}\left( \eta_{e} \right)} \right]$ (S12)

$S_{h}=\frac{k_{B}}{q}\left[ \frac{3}{2}\frac{F_{\frac{1}{2}}(\eta_{h})}{F_{-\frac{1}{2}}(\eta_{h})}-\left( r+\frac{5}{2} \right)\frac{F_{r+\frac{3}{2}}(\eta_{h})}{F_{r+\frac{1}{2}}(\eta_{h})} \right]$ (S13)

The boundary conditions at the two ends of the nanoribbon were: $V=0$, $n\cdot j_{e}=0$， $n\cdot j_{h}=0$, $n\cdot D=0$.

## Calculation of the lattice temperature distribution

The lattice temperature distribution and the corresponding voltage distribution in the Si nanoribbon, calculated by the multi-physics model, are shown in Fig. S2. The incident laser power was 0.1 μW (2 W cm^-2^). It was assumed that the absorbed photon energy all went to the lattice system and the heat transfer module was used. The simulated lattice temperature rise did not exceed 0.01 K, and the simulated maximum voltage difference was ~1 μV, which is five orders of magnitude smaller than the experimental value.


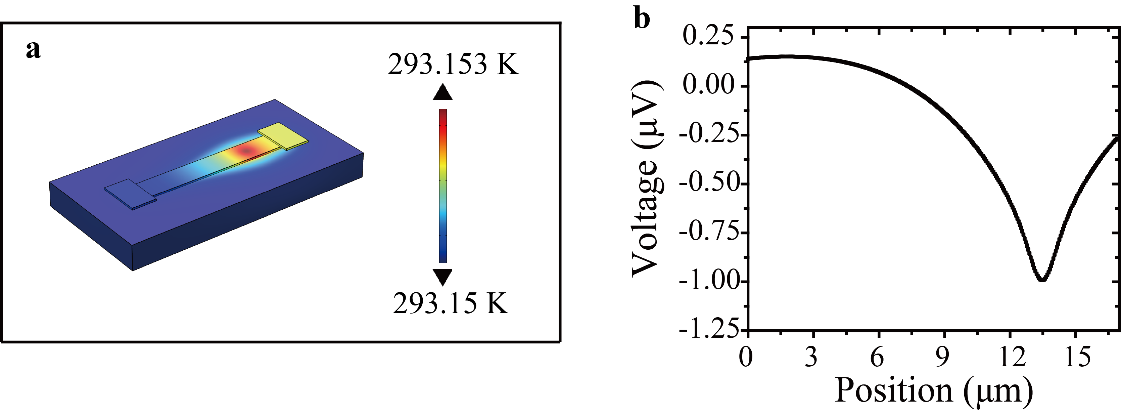


**Fig. S2. a** Simulated lattice temperature distribution in the Si nanoribbon. **b** Simulated voltage distribution along the Si nanoribbon.

## Calculation of the carrier thermal conductivity and heat capacity

The carrier thermal conductivity and the carrier heat capacity was calculated by

$\kappa_{c}=k_{B}^{2}N_{c}\mu_{c}\frac{T_{c}}{q}\frac{F_{0}\left( \eta_{c} \right)}{F_{\frac{1}{2}}\left( \eta_{c} \right)}\left[ \frac{6F_{2}\left( \eta_{c} \right)}{F_{0}\left( \eta_{c} \right)}-\frac{4F_{1}^{2}\left( \eta_{c} \right)}{F_{0}^{2}\left( \eta_{c} \right)} \right]$ (S14)

$C_{c}=\frac{3}{2}k_{B}\left[ n\frac{F_{r+\frac{3}{2}}\left( \eta_{e} \right)}{F_{r+\frac{1}{2}}\left( \eta_{e} \right)}+p\frac{F_{r+\frac{3}{2}}\left( \eta_{h} \right)}{F_{r+\frac{1}{2}}\left( \eta_{h} \right)} \right]$ (S15)

In the model, r = 0.5 was used as the effects of acoustic phonon scattering and strong shielded ion impurity scattering were mainly considered. In the range of temperature (293-670 K) and carrier concentration (10^18^ to 10^21^ m^-3^) considered in this model, the ratios of the Fermi-Dirac integrals were all close to unity. Therefore, the carrier thermal conductivity and heat capacity were simplified as:

$\kappa_{c}=\frac{k_{B}^{2}T_{c}}{q}\left( \mu_{e}n+\mu_{h}p \right)\text{×2}$ (S16)

$C_{c}=\frac{3}{2}k_{B}\left( n+p \right)$ (S17)

The electron and hole concentrations were estimated by the Boltzmann distribution at low doping concentration, with $E_{C}=-\text{4.05 eV}$, $E_{V}=-\text{5.17 eV}$, and $E_{f}=-\text{4.74 eV}$:

$n(T)=\int_{E_{C}}^{\infty} \frac{1}{2\pi^{2}}\left( \frac{2m_{e}^{*}}{\hbar^{2}} \right)^{\frac{3}{2}}\sqrt{E-E_{C}}\cdot e^{-\frac{E-E_{f}}{k_{B}T}}dE$ (S18)

$p(T)=\int_{-\infty}^{E_{V}} \frac{1}{2\pi^{2}}\left( \frac{2m_{p}^{*}}{\hbar^{2}} \right)^{\frac{3}{2}}\sqrt{E_{V}-E}\cdot e^{-\frac{E_{f}-E}{k_{B}T}}dE$ (S19)

Finally, the carrier thermal conductivity and heat capacity was calculated and the dependence on carrier temperature was plotted, as shown in Fig. S4. The carrier heat capacity and thermal conductivity increased rapidly with increasing carrier temperature. This may explain the saturation behavior of the carrier temperature and the $V_{\mathrm{oc}}$ saturation behavior with increasing laser power intensity.


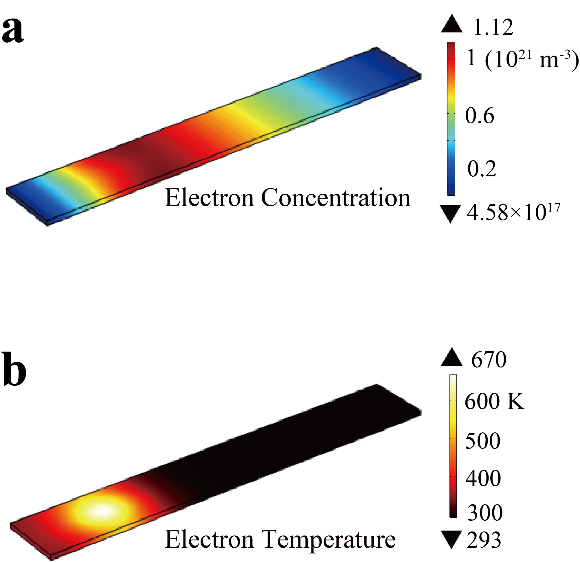


**Fig. S3.** Simulated spatial distribution of **a** electron concentration and **b** electron temperature in the Si nanoribbon at 16.2 W/cm^2^ laser power density.


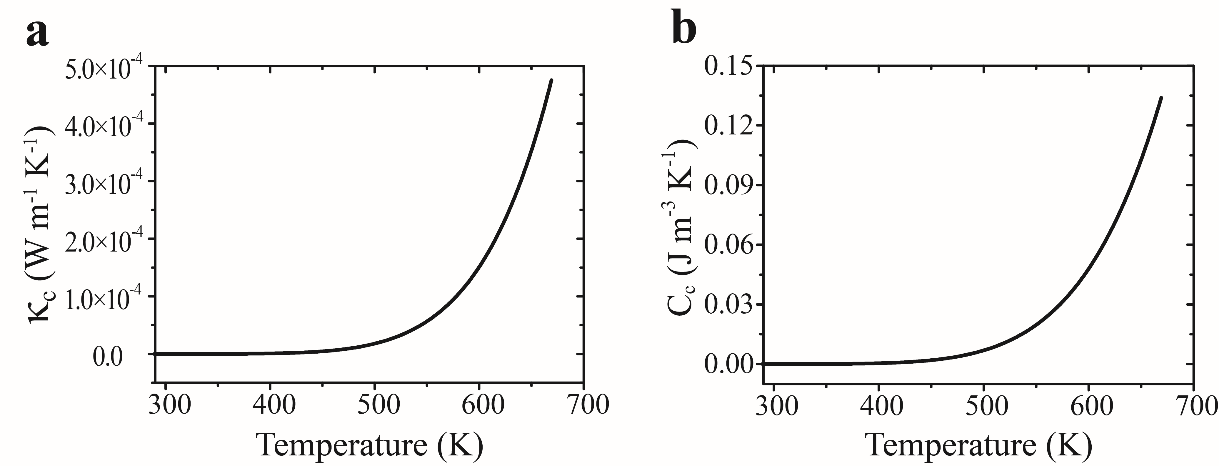


**Fig. S4.** The dependence of the **a** carrier thermal conductivity $\kappa_{c}$ and **b** carrier heat capacity $C_{c}$ on the carrier temperature.

### Some notes on the model

When the carrier concentration is less than 10^23^ m^-3^, according to the formula:

$\Delta E_{g}=E_{ref}\left( \ln\left( \frac{n}{N_{ref}} \right)+\sqrt{\left[ \ln\left( \frac{n}{N_{ref}} \right) \right]^{2}+\frac{1}{2}} \right)$ (S20)

the bandgap narrowing effect (<0.01 V) can be neglected. Here $E_{ref}=\text{0.00692 V}$, and $N_{ref}\text{=1.3×}\text{10}^{\text{23}}\text{ }\text{m}^{\text{-3}}$.

The discretized grid size in the semiconductor model must be smaller than the Debye length, $L_{d}=\sqrt{\frac{k_{B}T\varepsilon_{0}\varepsilon_{r}}{q^{2}N_{A}}}$, which is 3 μm at doping level 10^18^ m^-3^ and temperature 300 K in the model.

The carrier mobility model refers to the lattice scattering contribution and carrier scattering contribution:

$\frac{1}{u_{e}}=\frac{1}{u_{e0}}+\frac{1}{u_{cc}}$ (S21)

$\frac{1}{u_{h}}=\frac{1}{u_{h0}}+\frac{1}{u_{cc}}$ (S22)

$u_{cc}=\frac{\left( \frac{T}{T_{ref}} \right)^{\frac{3}{2}}F_{1}}{\left( np \right)^{\frac{1}{2}}\ln\left( 1+\left( \frac{T}{T_{ref}} \right)^{2}\left( np \right)^{-\frac{1}{3}}F_{2} \right)}$ (S23)

The electron and hole mobilities are $\mu_{e0}=\text{0.015} \text{m}^{\text{2}}\text{ }\text{V}^{\text{-1}}\text{ }\text{s}^{\text{-1}}$and $\mu_{h0}=\text{0.0045}\text{ }\text{m}^{\text{2}}\text{ }\text{V}^{\text{-1}}\text{ }\text{s}^{\text{-1}}$. $F_{1}=\text{1.04×1}\text{0}^{\text{2}\text{3}}\text{ }\text{m}^{\text{-1}}\text{ }\text{V}^{\text{-1}}\text{ }\text{s}^{\text{-1}}$, $F_{2}= \text{7.45×1}\text{0}^{\text{1}\text{6}}{\text{ }\text{m}}^{\text{-}\text{2}}$.

## Dynamic response


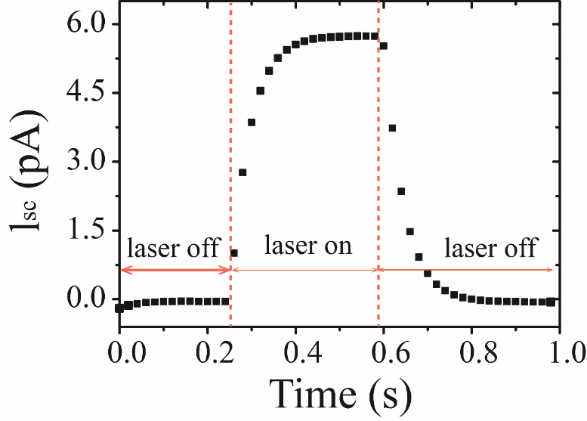


**Fig. S5.** The measured dynamic response of the $I_{\mathrm{sc}}$ with switching on/off of the incident laser.

**Table S1. The parameters used in the model**

| $k_{B}$ | Boltzmann constant | 1.38E- 23 | $\text{J}/\text{K}$ |
| --- | --- | --- | --- |
| q | Elementary charge | 1.60E- 19 | C |
| *h* | Planck constant | 6.63E- 34 | $\text{J s}$ |
| $\varepsilon_{0}$ | Vacuum dielectric constant | 8.85E- 12 | $\text{F }\text{m}^{\text{-1}}$ |
| c | Vacuum light speed | 3.00E+ 08 | $\text{m }\text{s}^{\text{-1}}$ |
| $E_{g}$ | Si band gap | 1.12 | eV |
| $\chi$ | Si affinity | 4.05 | eV |
| W | Chromium work function | 4.67 | eV |
| $m_{e}^{*}$ | Conduction effective mass of electron | $0.26 m_{e}$ |  |
| $m_{p}^{*}$ | Conduction effective mass of hole | $0.37 m_{e}$ |  |
| $\mu_{e0}$ | Electron mobility | 0.015 | $\text{m}^{\text{2}}\text{ }\text{V}^{\text{-1}}\text{ }\text{s}^{\text{-1}}$ |
| $\mu_{h0}$ | Hole mobility | 0.0045 | $\text{m}^{\text{2}}\text{ }\text{V}^{\text{-1}}\text{ }\text{s}^{\text{-1}}$ |
| $\kappa_{\text{Si}}$ | Si thermal conductivity | 131 | $\text{W }\text{m}^{\text{-1}}\text{ }\text{K}^{\text{-1}}$ |
| $C_{\text{Si}}$ | Si heat capacity | 1.63E +06 | $\text{J }\text{m}^{\text{-3}}\text{ }\text{K}^{\text{-1}}$ |
| $\kappa_{\text{SiO}_{\text{2}}}$ | Silica thermal conductivity | 1.38 | $\text{W }\text{m}^{\text{-1}}\text{ }\text{K}^{\text{-1}}$ |
| $C_{\text{SiO}_{\text{2}}}$ | Silica heat capacity | 1.55E +06 | $\text{J }\text{m}^{\text{-3}}\text{ }\text{K}^{\text{-1}}$ |
| $\varepsilon_{r}$ | Dielectric constant of Si | 15+ 0.113i |  |
| $\text{N}_{\text{A}}$ | Boron doping concentration | 1.72E+ 18 | $\text{m}^{\text{-3}}$ |
| $\beta$ | Single photon absorption coefficient | 1.021E+ 05 | $\text{m}^{\text{-1}}$ |
| $\gamma$ | Auger recombination coefficient | 3.8E- 43 | $\text{m}^{\text{6}}\text{ }\text{s}^{\text{-1}}$ |

## References:

1. Babar, S. & Weaver, J. H. Optical constants of Cu, Ag, and Au revisited. *Applied Optics* **54,** 477-481 (2015).
2. Palik, E. D. (ed) Handbook of Optical Constants of Solids II (Academic Press, 1991).
3. van Driel, H. M. Kinetics of high-density plasmas generated in Si by 1.06- and 0.53- μm picosecond laser pulses. *Physical Review B* **35,** 8166-8176 (1987).
4. Bulgakova, N. M. et al. A general continuum approach to describe fast electronic transport in pulsed laser irradiated materials: the problem of coulomb explosion. *Applied Physics A* **81,** 345-356 (2005).
5. Okuto, Y. & Crowell, C. R. Threshold energy effect on avalanche breakdown voltage in semiconductor junctions. *Solid-State Electronics* **18,** 161-168 (1975).
6. Klaassen, D. B. M. *et al*. Unified apparent bandgap narrowing in n- and p-type silicon. *Solid-State Electronics* **35,** 125-129 (1992).
7. Jain, S. C. & Roulston, D. J. A simple expression for band gap narrowing (BGN) in heavily doped Si, Ge, GaAs and GexSi1−x strained layers. *Solid-State Electronics* **34,** 453-465 (1991).
8. Gomes, C. J. *et al*. In-plane and out-of-plane thermal conductivity of silicon thin films predicted by molecular dynamics. *Journal of Heat Transfer* **128,** 1114-1121 (2006).
9. Jeong, C. *et al*. Thermal conductivity of bulk and thin-film silicon: a landauer approach. *Journal of Applied Physics* **111,** 093708 (2012).
10. Derrien, T. J. *et al*. Application of a two-temperature model for the investigation of the periodic structure formation on Si surface in femtosecond laser interactions. *Journal of Optoelectronics and Advanced Materials* **12,** 610-615 (2010).
